# Whole-Brain Mapping of Inputs to Projection Neurons and Cholinergic Interneurons in the Dorsal Striatum

**DOI:** 10.1371/journal.pone.0123381

**Published:** 2015-04-01

**Authors:** Qingchun Guo, Daqing Wang, Xiaobin He, Qiru Feng, Rui Lin, Fuqiang Xu, Ling Fu, Minmin Luo

**Affiliations:** 1 Britton Chance Center for Biomedical Photonics, Wuhan National Laboratory for Optoelectronics-Huazhong University of Science and Technology, Wuhan, China; 2 MoE Key Laboratory for Biomedical Photonics, Department of Biomedical Engineering, Huazhong University of Science and Technology, Wuhan, China; 3 National Institute of Biological Sciences, Beijing, China; 4 School of Life Sciences, Tsinghua University, Beijing, China; 5 Wuhan Institute of Physics and Mathematics, Chinese Academy of Sciences, Wuhan, China; Baylor College of Medicine, UNITED STATES

## Abstract

The dorsal striatum integrates inputs from multiple brain areas to coordinate voluntary movements, associative plasticity, and reinforcement learning. Its projection neurons consist of the GABAergic medium spiny neurons (MSNs) that express dopamine receptor type 1 (D1) or dopamine receptor type 2 (D2). Cholinergic interneurons account for a small portion of striatal neuron populations, but they play important roles in striatal functions by synapsing onto the MSNs and other local interneurons. By combining the modified rabies virus with specific Cre- mouse lines, a recent study mapped the monosynaptic input patterns to MSNs. Because only a small number of extrastriatal neurons were labeled in the prior study, it is important to reexamine the input patterns of MSNs with higher labeling efficiency. Additionally, the whole-brain innervation pattern of cholinergic interneurons remains unknown. Using the rabies virus-based transsynaptic tracing method in this study, we comprehensively charted the brain areas that provide direct inputs to D1-MSNs, D2-MSNs, and cholinergic interneurons in the dorsal striatum. We found that both types of projection neurons and the cholinergic interneurons receive extensive inputs from discrete brain areas in the cortex, thalamus, amygdala, and other subcortical areas, several of which were not reported in the previous study. The MSNs and cholinergic interneurons share largely common inputs from areas outside the striatum. However, innervations within the dorsal striatum represent a significantly larger proportion of total inputs for cholinergic interneurons than for the MSNs. The comprehensive maps of direct inputs to striatal MSNs and cholinergic interneurons shall assist future functional dissection of the striatal circuits.

## Introduction

As the key input station in the basal ganglia, the dorsal striatum coordinates neuronal signals from multiple cortical and subcortical regions and plays a critical role in voluntary movements, motor learning, cognition, and emotion [1,2,3,4]. D1- or D2-expressing medium spiny neurons (MSNs) are projection neurons and consist of ~90% of the total striatal neuron population [5,6]. D1 MSNs project to the internal globus pallidus (GPi)/entopeduncular nucleus (EP) and the substantia nigra pars reticulata (SNr), which forms the direct pathway [5,6]. D2-type MSNs indirectly activate the GPi-SNr complex through the external globus pallidus (GPe) and the excitatory subthalamic nucleus, thereby forming the indirect pathway. The direct and indirect pathways have been theorized to provide antagonistic feedback signals to the cortex and serve as an action selector to choose behaviors from many possible ones [7,8]. Dysfunction of the basal ganglia system is associated with multiple neurological disorders, such as Parkinson's disease and Huntington's disease [9,10].

Cholinergic interneurons consist of approximately 2% of total neurons in the dorsal striatum [6]. Although they are sparsely distributed, studies have provided a growing body of evidence that cholinergic interneurons are involved in the important functions of associative learning, reward processing, and motor control [11]. Tonically active neurons (TANs), presumed to be striatal cholinergic neurons, show fast regular spontaneous activity and respond to motivationally salient stimuli with a pause and subsequent rebound increase in their firing activity [12,13,14,15,16]. They also exhibit pronounced excitation during animal movements [17]. This activity is closely correlated with the phasic activity of dopamine neurons and contributes to the presynaptic regulation of dopamine and GABA release from neurons in the substantia nigra pars compacta (SNc) [18,19,20,21].

Identification of the inputs to various types of striatal neurons is essential for understanding how neuronal activity patterns are shaped. Using the traditional method of tract tracing, numerous studies have shown that the dorsal striatum receives converging inputs from the cortex, thalamus, SNc, and other subcortical nuclei [22,23,24,25,26,27]. Electron microscopy studies and physiological recordings have shown that neurons in many of these input areas connect with both MSNs and striatal cholinergic neurons [28,29]. However, these traditional techniques are unsuitable for mapping the whole-brain inputs to specific neuron types. The development of rabies virus-based retrograde tracing methods [30,31,32,33,34] together with numerous Cre-expressing mouse lines has provided powerful tools for studying the distribution patterns of neurons that provide monosynaptic inputs to genetically identifiable neuron populations. Using this method, Wall et al. recently mapped the direct inputs to D1 and D2 MSNs [35]. However, this study labeled only a small number (~200) of input neurons outside the striatum. Moreover, some brain structures that are known to project to the dorsal striatum were not revealed [36]. It remains unclear from the Wall et al. study whether neurons in the omitted structures do not form synapses with MSNs or the omission was the result of low labeling efficiency. Additionally, the input patterns for the cholinergic interneurons have not been studied at the whole-brain level using the transsynaptic tracing method. This mapping is particularly challenging for traditional tract tracing due to the sparse distribution of cholinergic interneurons throughout the dorsal striatum.

In this study, we studied the whole-brain input patterns of D1- and D2- MSNs and cholinergic interneurons by selectively infecting these neurons with the modified rabies virus. Our results reveal that the two types of MSNs and the cholinergic interneurons receive extensive inputs from discrete areas in the cortex, thalamus, amygdala, globus pallidus, and SNc. Moreover, cholinergic interneurons receive particularly dense local innervations from non-cholinergic neurons within the dorsal striatum.

## Materials and Methods

All procedures were conducted with the approval of the Institutional Animal Care and Use Committee (IACUC) of National Institute of Biological Sciences (NIBS), Beijing, China and the Ethics Committee of Wuhan Institute of Physics and Mathematics, Chinese Academy of Sciences.

### Animals

The BAC-transgenic Chat-Cre [MMRRC Tg(Chat-cre)24Gsat], D1-Cre [MMRRC Tg(Drd1a-cre)EY262Gsat], and D2-Cre [MMRRC Tg(Drd2-cre)ER44Gsat] mouse lines were acquired from MMRRC (Davis, California) [37]. These mice were back crossed and maintained in a C57BL/6J background and housed in groups on a 12-h light/12-h dark cycle with food and water *ad libitum*. Adult mice of either sex (8–16 weeks old) were used for all experiments, whereas age-matched wild-type C57BL/6J mice were used as the control.

### Viral vector preparation

The modified rabies virus and its helper adeno-associated virus (AAV) virus were constructed for monosynaptic retrograde tracing. The initial EnvA-pseudotyped, G-deleted rabies viruses (SADΔG-mCherry) and the cell lines for rabies propagation and titering were kindly supplied by E.M. Callaway at Salt Institute. The rabies viruses were produced and concentrated as previously described [32]. The final titer of SADΔG-mCherry was 10^8^ infecting unit per milliliter. The helper viruses included AAV with a DIO sequence carrying TVA (AAV-DIO-EGFP–TVA) or rabies glycoprotein (AAV-DIO-RG). The AAV-DIO-EGFP-TVA plasmid was constructed by subcloning the CAG promoter from the AAV-CAG-GFP-IRES-CRE plasmid (Addgene Plasmid 48201) and the coding region of EGFP:2A:TVA from the pAAV-EF1a-FLEX-GT plasmid (a gift from E.M. Callaway; Addgene plasmid 26198) into the DIO cassette of the plasmid pAAV-EF1a-DIO-hChR2(H134R)-EYFP (a gift from K. Deisseroth; Addgene plasmid 20298) [31,38]. Similarly, the AAV-DIO-RG plasmid was constructed by subcloning the CAG promoter from the AAV-CAG-GFP-IRES-CRE plasmid and coding region of RG from AAV-EF1a-FLEX-GTB (a gift from E.M. Callaway; Addgene plasmid 26197) into the DIO cassette of the plasmid pAAV-EF1a-DIO-hChR2(H134R)-EYFP [31,38]. AAV-DIO-EmGFP virus was used for axonal tracing. The AAV-DIO-EmGFP plasmid was constructed by replacing the coding region of hChR2(H134R)-EYFP in the pAAV-EF1a-DIO-hChR2(H134R)-EYFP plasmid with the coding sequence of enhanced membrane GFP (EmGFP; a gift from C. Cepko: Addgene plasmid 14757) [39]. All recombinant AAV viruses were packaged into 2/9 serotype at NIBS, Beijing, with final titers at 1–5 × 10^12^unit/ml.

### Animal surgery and virus injection

Before viral injection, the mouse was anesthetized with pentobarbital (i.p., 80 mg/kg). We then positioned the mouse in a custom designed stereotaxic instrument and kept it warm with a heating blanket. After disinfection with 0.3% hydrogen peroxide, a small incision of the scalp was created to expose the skull. For viral injection, a small 1-mm diameter hole in the skull was drilled 0.5 mm posterior to the bregma and 1.75 mm lateral to the midline. Mixtures of helper virus (AAV-DIO-EGFP-TVA and AAV-DIO-RG; 200 nL) were injected into the dorsal striatum (0.5 mm posterior to bregma, 1.75 mm lateral to the midline, 2.25 mm deep from dura) using a Nanoliter 2000 at a speed of 40 nL/min with a pulled glass pipette (Sutter Instrument). The injection pipette was withdrawn 10 min after the completion of virus injection. The incision was sealed with tissue adhesive (3M Vetbond 1469SB) immediately after pipette removal. The mouse was placed in a clean cage for recovery and AAV expression. Two weeks later, SADΔG-mCherry rabies virus (300 nL) was injected into the identical brain location under biosafety level 2 conditions with the same procedure mentioned above.

### Histology and immunostaining

One week after the injection of SADΔG-mCherry rabies virus injection, mice were sacrificed with an overdose of pentobarbital (i.p., 300 mg/kg) and then intracardially perfused with 0.9% saline solution followed by 4% paraformaldehyde (PFA) in PBS. Mouse brains were placed in 4% PFA solution overnight for post fixation and then 30% sucrose solution (2 days) for cryoprotection. Either coronal or sagittal sections (50 μm) were prepared with a freezing cryostat (Leica CM1900). Brain sections were mounted on chrome-gelatin subbed glass slides and cover slipped using 50% DAPI-glycerol mounting medium. For the immunohistochemistry, several coronal sections near the injection site were selected and stored in PBS solution. The sections were first blocked with 3% BSA in PBS-0.3% Triton X-100 for 5 minutes and incubated with the primary goat anti-ChAT antibody (1:200, Millipore AB144P) overnight at 4°C. After washing, the sections were incubated with fluorophore-conjugated anti-goat secondary antibody (1:500, Jackson ImmunoResearch) for 1 hour.

### Imaging and analysis

Images were acquired with a digital slide scanner (Axio Scan.Z1, Zeiss). DAPI channel was set as the reference channel to locate focal plane and a 10× objective was used for imaging. Three pseudocolor channels (red, green, and blue) were used for mCherry, EGFP and DAPI, respectively. Global brightness and contrast were adjusted to reduce background. For analysis, single brain sections were segmented with Fiji (National Institutes of Health, Bethesda, MA, USA). The locations of labeled neurons were manually registered and the labeling density was quantified with Fiji according to a standard mouse brain atlas [40]. Immunoreactivity signals were acquired with a Nikon A1 confocal microscope.

## Results and Discussion

**Fig 1:**
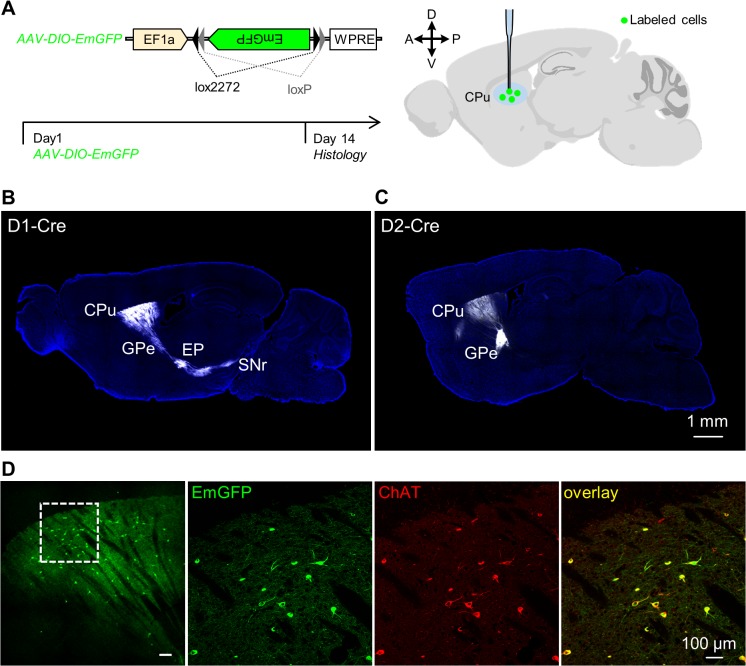
Validation of Chat-Cre, D1-Cre, and D2-Cre mouse lines. **(A)** Experimental design for axonal tracing. AAV-DIO-EmGFP virus was injected into the dorsal striatum of ChAT-Cre, D1-Cre, or D2-Cre mice. Histology was performed two weeks later. **(B)** Following the injection of AAV-DIO-EmGFP virus into the dorsal striatum of a D1-Cre mouse, we observed strong terminal fluorescent signals in the GPi/EP and the SNr. For clarity, EmGFP signals are shown in white. **(C)** AAV-DIO-EmGFP virus injection into the dorsal striatum of a D2-Cre mouse produced EmGFP+ terminal signals mainly in the GPe. **(D)** Infusion of AAV-DIO-EmGFP viral vectors in the dorsal striatum of a ChAT-Cre mouse resulted in EmGFP expression (green) exclusively in ChAT-immunopositive (red) neurons. Abbreviations: GPe, external globus pallidus; GPi/EP, internal globus pallidus/entopeduncular nucleus; SNr, substantia nigra, reticular part.

We first confirmed the accuracy of using D1-Cre, D2-Cre, and ChAT-Cre mouse lines to genetically target striatal D1 MSNs, D2 MSNs, and cholinergic interneurons, respectively. Following stereotaxic infusion of the Cre-dependent AAV-DIO-EmGFP vectors into the D1-Cre mice (Fig 1A), we observed dense EmGFP labeling in the dorsal striatum as well as the EP and the SNr, the major targets of D1 MSNs in the direct pathway [5,6] (Fig 1B). In D2-Cre mice, extrastriatal terminal signals were mainly found in the GPe, the primary target of D2 MSNs [5,6] (Fig 1C). Injections of AAV-DIO-EmGFP vectors in the dorsal striatum of ChAT-Cre mice resulted in strong EmGFP expression in a sparse set of striatal neurons that were always immunopositive to choline acetyltransferase (ChAT; Fig 1D). Consistent with the nature of interneurons, EmGFP-labeled terminals were restricted within the dorsal striatum [41]. These results therefore demonstrate that the D1-Cre, D2-Cre, and ChAT-Cre mouse lines could be used to drive gene expression specifically in D1 MSNs, D2 MSNs, and cholinergic interneurons.

**Fig 2:**
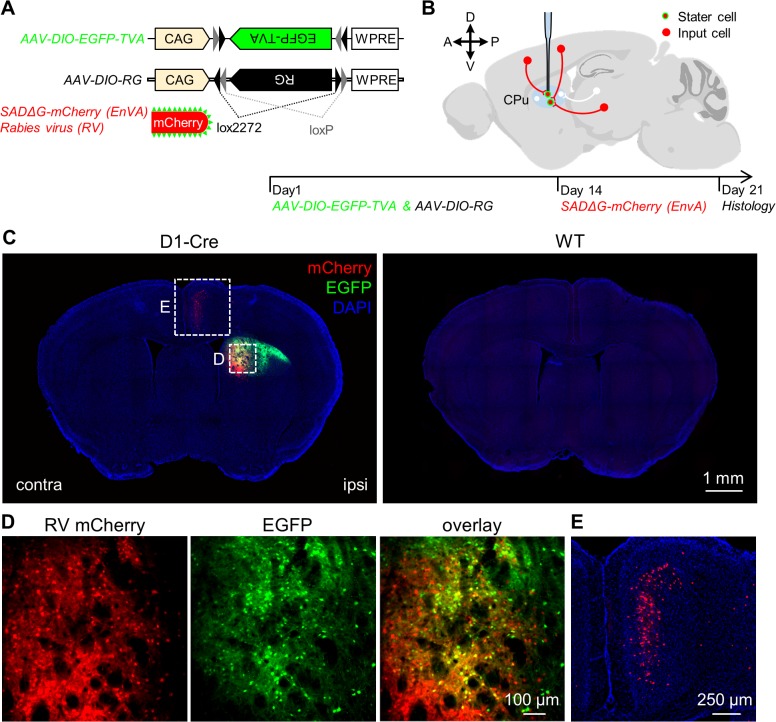
The method of rabies-based retrograde tracing. **(A)** AAV virus and rabies virus for transsynaptic retrograde tracing. Double-floxed EGFP-TVA and rabies glycoprotein (RG) under control of CAG were respectively packaged into AAV viral vectors. The rabies virus without glycoprotein (SADΔG) was equipped with the mCherry fluorescent protein. **(B)** Experimental design. On Day 1, AAV-DIO-EGFP-TVA and AAV-DIO-RG viral vectors were injected into the dorsal striatum of ChAT-Cre, D1-Cre, or D2-Cre mice. Two weeks later, SADΔG-mCherry was injected into the same site of the dorsal striatum. Histology was performed after one more week. The starter cells are labeled both in green and red, whereas the input neurons are labeled only in red. **(C)** Confirmation of effective transsynaptic labeling using rabies virus. Sequential injections of AAV-DIO-EGFP-TVA, AAV-DIO-RG and SADΔG-mCherry viruses into the dorsal striatum of a D1-Cre mouse labeled starter cells (green) and input neurons (red; left panel). Same virus injections did not label any neurons in a wild-type C57BL/6J mouse (right panel). **(D)** Zoom-in view of starter cells and transsynaptically labeled cells in the dorsal striatum of the D1-Cre mouse shown in (C). **(E)** Transsynaptic retrograde labeling neurons in the cingulate cortex (cg).

We combined modified rabies virus and its helper AAV viruses for transsynaptic retrograde tracing (Fig 2A and 2B) [34,35]. The rabies virus (SADΔG-mCherry) was modified by replacing the native rabies glycoprotein in the viral membrane with an avian sarcoma leucosis virus envelope protein (EnvA). Helper virus included two Cre-dependent AAV vectors that carried double-floxed inverted open reading frames of EGFP-TVA (AAV-DIO-EGFP-TVA) and rabies glycoprotein (AAV-DIO-RG), respectively (Fig 2A). The mixture of the two helper viruses (200 nL) was first injected into the dorsal striatum of individual Cre-expressing mice and C57BL/6J wild-type mice. Two weeks later, the modified rabies virus (300 nL) was injected into the same site. Mouse brains were sectioned and imaged one week later. AAV-DIO-EGFP-TVA viral vectors enabled the selective expression of TVA in Cre-expressing neurons and guided the infection of rabies virus. AAV-DIO-RG expressed rabies glycoprotein for retrograde spread of rabies virus from starter neurons to their directly presynaptic neurons. Further spread of rabies virus was stopped due to the absence of TVA and rabies glycoprotein in the neurons that were further upstream. Because the expression of TVA helper virus and modified rabies virus were separately tagged with EGFP and mCherry, respectively, the “starter” cells were labeled with both EGFP and mCherry, whereas the input neurons were labeled with mCherry only (Fig 2B).

Fig 2C shows the labeling patterns near the injection sites following unilateral injections of helper AAV viruses and the modified rabies virus into the dorsal striatum of a D1-Cre mouse and a wild-type mouse. Near the injection site in the D1-Cre mouse, we observed numerous EGFP+ neurons that also expressed mCherry, suggesting that they were starter cells (Fig 2C and 2D). Within the same coronal sections, we also observed dense distribution of mCherry-expressing neurons in the cingulate cortex, one of the known input areas of the dorsal striatum (Fig 2C and 2E). By contrast, we did not detect the presence of either EGFP or mCherry signals in the striatum or the cingulate cortex of the control C57BL/6J mice that received the same treatment of virus injection (Fig 2C). These results indicate that extrastriatal mCherry labeling was produced by the monosynaptic spread of rabies virus from Cre-expressing neurons within the striatum.

**Fig 3:**
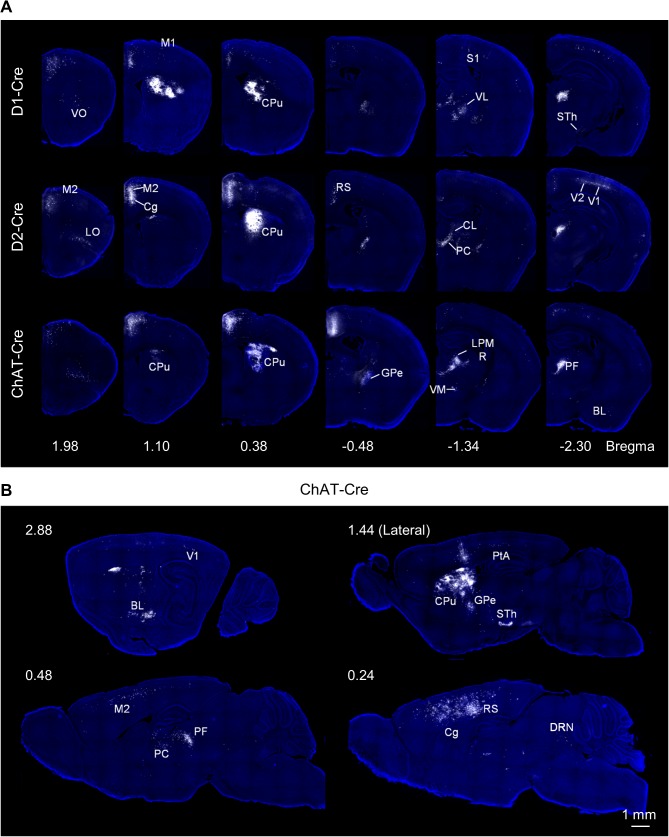
Monosynaptic inputs onto D1, D2, and ChAT neurons in the dorsal striatum. **(A)** Coronal representation of retrograde monosynaptic labeling of input neurons onto D1 MSNs, D2 MSNs, and cholinergic interneurons. **(B)** Sagittal brain sections of an infected ChAT-Cre mouse, showing innervations to cholinergic neurons in the dorsal striatum. Abbreviations: Au, auditory cortex; BL, Basolateral amygdaloid nucleus; Cg, cingulate cortex; CL, centrolateral thalamic nucleus; CPu, caudate putamen; DRN, dorsal raphe nucleus; GPe, external globus pallidus; LO, lateral orbital cortex; LPMR, lateral posterior thalamic nucleus, mediorostral part; M2, secondary motor cortex; PC, paracentral thalamic nucleus; PF, parafascicular thalamic nucleus; PtA, parietal association cortex; RS, retrosplenial cortex; S1, primary somatosensory cortex; STh, subthalamic nucleus; VO, ventral orbital cortex; V1, primary visual cortex; V2, secondary visual cortex; VL, ventrolateral thalamic nucleus.

We injected helper AAV viral vectors and then modified rabies virus into the center of the unilateral dorsal striatum of all three Cre-expressing mouse lines (n = 4 mice for each line). Fig 3A shows the mCherry labeling pattern within the representative coronal sections of D1-Cre, D2-Cre, and ChAT-Cre mice, respectively. Fig 3B displays representative sagittal brain sections from a ChAT-Cre mouse. Input neurons labeled with mCherry signals were presented using the white pseudo-color to highlight the precise locations in a blue background of DAPI counterstaining of cell nuclei. Anatomical structures were identified according to a standard mouse atlas [40]. For the three striatal neuron types examined, extensive retrograde labeling was present in many discrete areas in the ipsilateral hemisphere, although sparser labeling was also observed in the contralateral hemisphere (data not shown).

**Fig 4:**
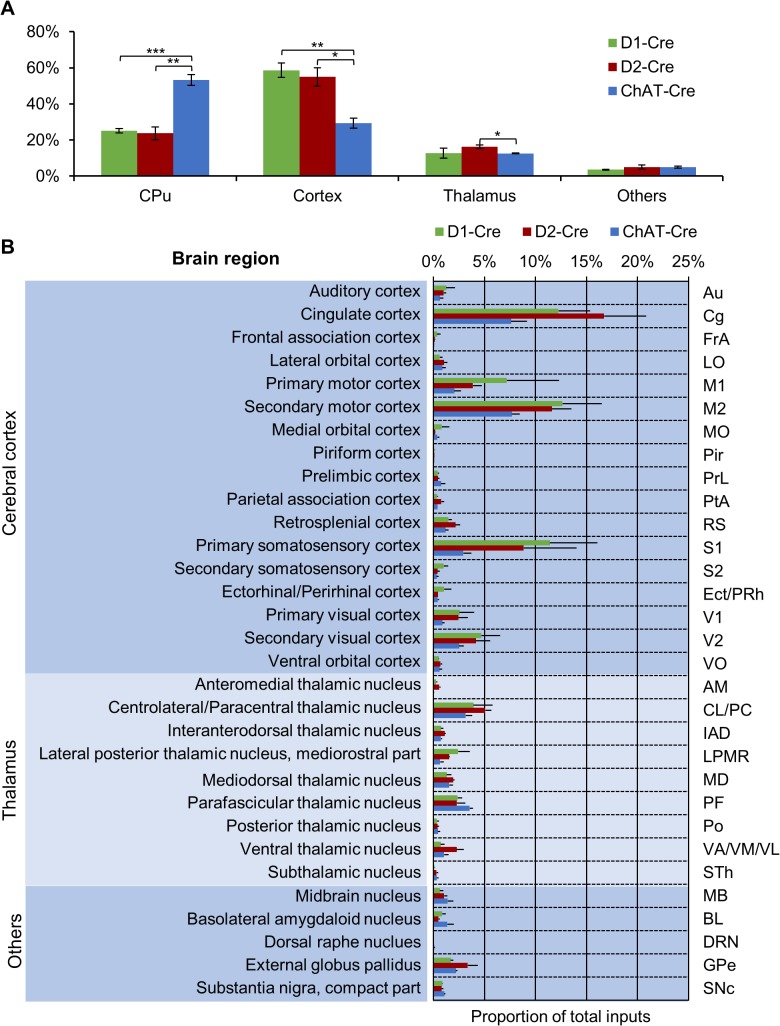
Quantitative analysis of whole-brain monosynaptic inputs to the dorsal striatum D1, D2, and ChAT neurons. **(A)** We clustered input area into the dorsal striatum, cortex, thalamus, and other nuclei. Proportions of input neurons from the dorsal striatum and the cortex are significantly different between D1/D2 projection neurons and cholinergic interneurons. Error bars indicate SEM. *p < 0.05, **p < 0.01, ***p < 0.001, two tailed t-test; n = 4 mice. **(B)** Proportion of specific extrastriatal areas for each striatal cell type.

We quantified the number of mCherry-expressing neurons in individual nuclei within the entire brain of each mouse and then normalized the cell numbers by the total number of input neurons within each animal to correct for the variability in the numbers of starter cells (Fig 4). In both the D1- and D2-Cre mice, we detected over 7,000 clearly labeled neurons, most of which were located outside the striatum (n = 5620 ± 2809 or 74.9% are extrastriatal neurons for D1-Cre mice; n = 5532 ± 2846 or 76.2% for D2-Cre; Mean ± SD; Fig 4A). In ChAT-Cre mice, we found nearly 5,000 retrograde labeled neurons, slightly less than half of which were outside the striatum (n = 2318 ± 545 or 46.7% neurons outside the striatum). For all three neuron types, the majority of the external input neurons were located in the cortex and thalamus (Fig 4A). Over half of the total MSN inputs arose from corticostriatal neurons (58.7% ± 6.8% for D1 neurons; 54.9% ± 8.8% for D2 neurons), whereas approximately one-third of input neurons came from the cortex for the striatal cholinergic interneurons (29.3% ± 4.8%; Fig 4A). Inputs from the thalamus occupied ~10% of total inputs for the MSNs and cholinergic interneurons, with the remaining extrastriatal input areas contributing to less than 5% of total inputs for both MSNs and cholinergic interneurons.

**Fig 5:**
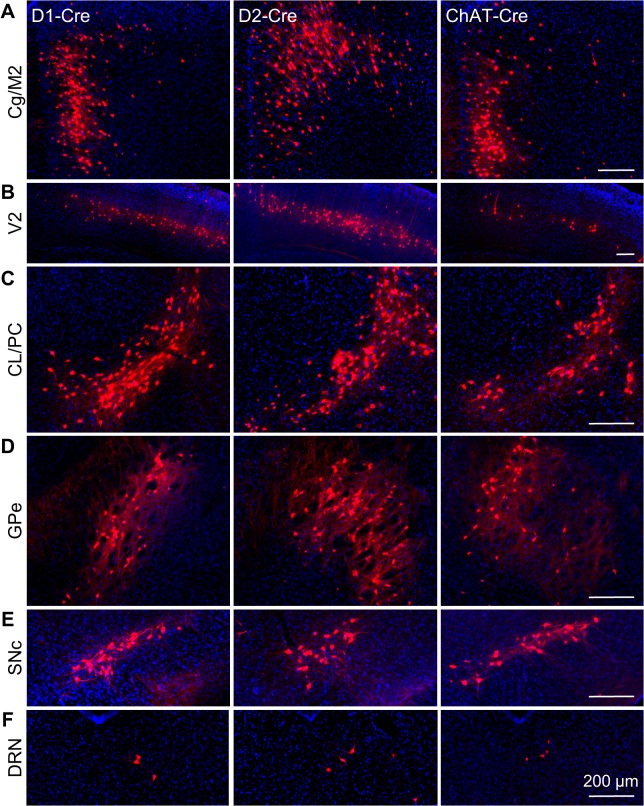
Strong retrograde labeling in representative brain areas following rabies virus infection of striatal D1 MSNs, D2 MSNs, and cholinergic interneurons. (**A**) The cingulate cortex and secondary motor cortex (Cg/M2). (**B**) The secondary visual cortex (V2). (**C**) The centrolateral/paracentral thalamic nucleus (CL/PL). (**D**) The external globus pallidus (GPe). (**E**) The substantia nigra compacta (SNc). (**F**) The dorsal raphe nucleus (DRN).

Fig 4B displays the proportion of input neurons in specific regions. The overall distribution patterns for D1 MSNs and D2 MSNs were overall similar, although cortical inputs tended to make fewer contributions to cholinergic interneurons. The ipsilateral somatosensory cortex, motor cortex, cingulate cortex, and visual cortex comprised the vast majority of cortical inputs (Figs 4B and 5A and 5B). We observed much less labeling in the auditory cortex and the piriform cortex. In the thalamus, strong labeling was found in the centrolateral/paracentral thalamic nucleus (CL/PC) and the parafascicular thalamic nucleus (Figs 4B and 5C). We also found substantial retrograde labeling in the basolateral amygdaloid nucleus, the GPe, and the SNc (Figs 4B and 5D and 5E). It is worth mentioning that the Wall et al. study reported scarce or no labeling in the cingulate cortex, visual cortex, CL/PC, GPe, and SNc [35], which contained substantial labeling in this study (Fig 5A–5E). We observed only a few retrograde labeled neurons in the dorsal raphe nucleus (DRN; Fig 5F).

**Fig 6:**
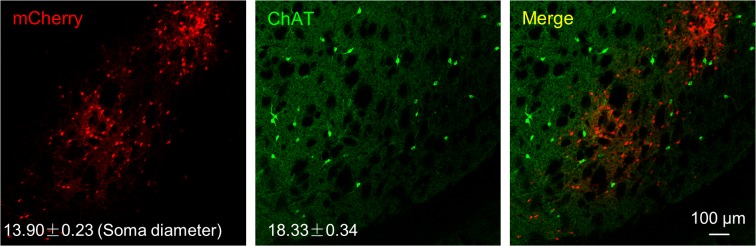
The majority of the intrastriatal input neurons targeting the ChAT neurons are non-cholinergic. Following the injection of rabies virus and its helper virus, retrograde labeled neurons (red) and cholinergic neurons (green) did not overlap. Additionally, the intrastriatal input neurons and ChAT neurons exhibited distinct morphology and soma sizes.

Due to the strong intrastriatal innervation of dorsal striatal cholinergic neurons, we asked whether the striatal input neurons were cholinergic. Following virus injections into ChAT-Cre mice, we labeled dorsal striatal cholinergic neurons by immunostaining against ChAT (Fig 6). We did not detect any co-localizations of mCherry signal and ChAT immunoreactivity within the striatum. Furthermore, a clear morphological difference existed between retrogradely labeled, mCherry-expressing neurons and ChAT-immunopositive neurons. The average soma size of the intrastriatal input neurons (13.90 ± 0.23 μm; mean ± SEM, n = 100 cells) was significantly smaller than that of ChAT-expressing neurons (18.33 ± 0.34 μm; n = 27 cells; p = 9.9 × 10^–16^, two-tailed t-test), which further suggests that the intrastriatal input neurons were non-cholinergic.

**Fig 7:**
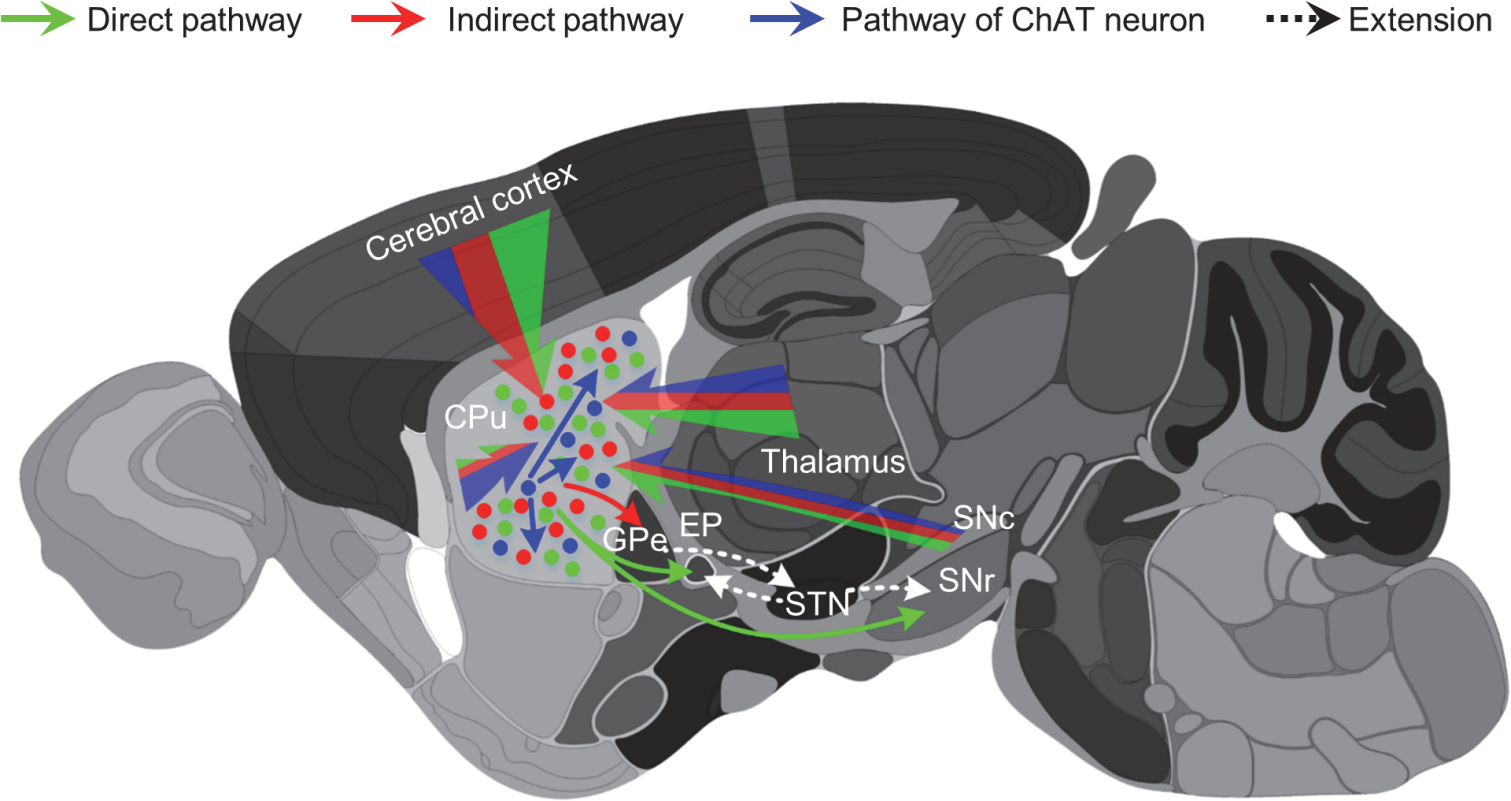
Summary of the inputs to the dorsal striatum cholinergic interneurons and D1/D2 projection neurons. Blue, green, and red lines indicate monosynaptic inputs to striatal ChAT, D1, and D2 neurons, respectively. Thickness of the lines represents proportional input strength (cell numbers) from given brain areas. Abbreviations: CPu, caudate putamen; GPe, external globus pallidus; GPi/EP, internal globus pallidus/entopeduncular nucleus; SNc, substantia nigra, compact part; SNr, substantia nigra, reticular part; STN, subthalamic nucleus;.

Fig 7 summarizes the input patterns of the two types of MSNs and cholinergic interneurons. Consistent with the prior transsynaptic tracing [35], we found that striatal D1- and D2-type projection neurons received direct inputs mainly from the cortex and the thalamus. These input neurons tended to cluster in the motor cortex, somatosensory cortex, mediodorsal thalamic nucleus, and parafascicular thalamic nucleus. However, two major differences should be noted between this and the prior transsynaptic tracing. First, Wall et al. detected ~200 retrograde labeled neurons per animal outside of the striatum, whereas over 5,000 neurons were labeled in this study. This difference may result from differences in the injected volume of rabies (180 nL by Wall et al. *vs*. 300 nL in this study) and/or variability in effective titers of rabies virus. The labeling density in this study is more consistent with that revealed by many previous retrograde tracings. Most likely, striatal MSNs receive much more extensive inputs than what can be inferred from the number of ~200 of retrograde labeled neurons.

Second, we observed substantial retrograde labeling in the secondary visual cortex, cingulate cortex, centrolateral/paracentral thalamic nucleus, GPe, and SNc, whereas the labeling within these areas was very modest or absent in the prior study [35]. Our data are consistent with the previous retrograde trace studies using traditional tract tracers [42,43,44,45,46]. The cingulate cortex is a key node in the limbic system that processes emotional and motivational signals [47]. The centrolateral/paracentral thalamic nucleus is part of the intralaminar thalamus that is involved in sensory and motor functions [43,48]. The SNc is the key source of dopamine for the dorsal striatum [49]. Therefore, MSNs and cholinergic neurons integrate inputs from extensive brain areas that are related to sensory (somatosensory and visual), motor, emotional/motivational, and modulatory (dopamine) functions. They also receive feedback signals from the GPe [45,46]. It had be speculated that weak transsynaptic labeling results from the non-synaptic connections or the nature of peculiar synapses, which prevent the rabies virus from spreading from the striatal neurons to the input neurons [35]. For example, the very weak labeling in the SNc might reflect the possibility that dopamine neurons in the SNc do not form classic synapses with MSNs [35]. Although the presence of specialized synaptic connections cannot be excluded, our observation of strong labeling in the SNc suggests that the modified rabies virus particles can effectively traverse the connecting structures between dopamine axons and striatal neurons including MSNs and cholinergic interneurons. However, some tract tracing studies show substantial retrograde labeling in the DRN following the deposition of tracers in the striatum [50]. We only observed weak labeling in the DRN. Axonal labeling showed that DRN 5-HT neurons project dense axonal terminals to the ventral but not dorsal striatum [51], suggesting that it is unlikely that the dorsal striatum receives heavy inputs from the DRN.

We found that the striatal cholinergic interneurons received much stronger intrastriatal innervation than the MSNs, which is consistent with the role of cholinergic interneurons in intrastriatal processing [18,38,49,52,53,54]. Similar to MSNs, cholinergic interneurons also receive extensive inputs from the cortex, thalamus, amygdala, GPe, and SNc. Previous studies have emphasized the physiological effect of dopaminergic inputs from the SNc on cholinergic neurons [18,20]. The extensive distal innervation suggests that the inputs from a much broader set of brain areas are integrated by cholinergic interneurons before they send output to influence other striatal neurons.

Comprehensive maps of monosynaptic input patterns for MSNs and cholinergic neurons should facilitate dissection of physiological and behavioral functions of various afferents of the dorsal striatum. The dorsal striatum contains additional interneurons, including the GABA/parvalbumin fast spiking interneurons and GABA/somatostatin/nitric oxide interneurons [55]. The inputs to these interneurons can also be investigated with the rabies virus-based transsynaptic tracing technique to complete the maps for all major neuron types in the striatum. Because cortical projections to the dorsal striatum are organized with broad topography, it is possible that different input patterns could be revealed with more complete sampling of injection sites. Finally, the spread of rabies virus may not be tightly correlated with synaptic weights. The physiological significance of the retrograde maps can only be unraveled by systematic functional studies.

## Conclusions

In this study, we mapped the distribution of input neurons to D1- and D2-expressing projection neurons and ChAT-expressing interneurons in the dorsal striatum using transsynaptic retrograde tracing with a modified rabies virus. All three types of neurons receive extensive inputs from cortical and thalamic areas that are involved in sensory and motor processing. They also receive input from limbic areas such as the cingulate cortex and amygdala. Additional notable input areas include the GPe and the SNc. Distal innervation patterns are similar for the two types of MSNs and the cholinergic interneurons. However, cholinergic interneurons receive denser intrastriatal inputs from the non-cholinergic neurons within the dorsal striatum.
